# The Relationship between Paranoid Tendencies and Cyberbullying among Chinese Adolescents: The Mediating Role of Moral Disengagement

**DOI:** 10.3390/bs13020102

**Published:** 2023-01-26

**Authors:** Shuangjia Lin, Bin Xiao

**Affiliations:** 1 School of Educational Sciences, Bohai University, Jinzhou 121013, China; 2 School of Humanities and Law, Northeastern University, Shenyang 110819, China

**Keywords:** paranoid tendencies, cyberbullying, moral disengagement, adolescents

## Abstract

BACKGROUND: Cyberbullying has become an essential public health psychological issue affecting people’s lives in the online ecology. However, previous studies have rarely examined adolescent paranoia, moral cognition, and cyberbullying in association. Therefore, this study was based on cognitive-behavioral theory to investigate the relationship between child-like paranoid tendencies, cyberbullying, and moral disengagement. METHODS: This study used the Paranoia Scale, Cyberbullying Scale, and Moral Disengagement Scale to conduct an anonymous online survey of 1519 adolescents in China. RESULTS: (1) Paranoid tendencies, moral disengagement, and cyberbullying were all significantly and positively correlated. Boys showed higher rates of moral disengagement, while girls showed higher rates of paranoid tendencies. (2) The direct effect of paranoid tendency on cyberbullying was significant (β = 0.31, *p* < 0.01). (3) There was a mediating effect of moral disengagement in the influence of paranoid tendencies on cyberbullying, with an effect proportion of 20.5%. CONCLUSION: Adolescent cyberbullying should be regulated at the family and social levels to enhance juvenile mental health issues and help them establish proper moral standards.

## 1. Introduction

In the age of cyber technology, cyberbullying has become an essential public health psychological issue affecting people’s lives in the cyber ecology. While the Internet allows adolescents to quickly access information and expand their horizons, it also inevitably affects their cognition, emotions, attitudes, and behaviors. The Internet’s influence can also be harmful due to the accessibility, anonymity, and secrecy of the online environment. Cyberbullying is an important social issue arising from this.

According to Olweus, cyberbullying is an intentional and repetitive act of causing harm to others using electronic messages as a medium [1]; Slonje argues that cyberbullying is an act of aggression by an individual or group that is repeatedly produced against individuals who cannot easily protect themselves through the use of electronic message communication [2]; Waasdorp defines cyberbullying as an individual or group of people who engage in malicious, repetitive [3], and hostile behaviors against others with the help of communication technology and can cause harm to others [4]. Based on the definition of scholars, it can be seen that cyberbullying is a repetitive, aggressive, and harmful behavior based on the online environment [5], in the form of text messages, web posts, emails, or other electronic communications, committed by a strong party against a weak party. In recent years, scholars have mainly focused on two aspects of cyberbullying: the motives and characteristics of cyberbullying [6]. On the one hand, some scholars point out that the motivation for cyberbullying by adolescents is retaliatory behavior for traditional bullying suffered offline [7]. Some adolescents inflict cyberbullying on others to experience a sense of power [8]. On the other hand, some scholars point out that the characteristics of the Internet include ambiguity [9], anonymity, complexity, and universality [7]. Because of these characteristics, adolescents may ignore moral consciousness, lack ethical norms, and ignore the consequences of their words and actions in online behavior, thus exacerbating cyberbullying among adolescents. Additionally, based on cognitive-behavioral theory, under the cognitive–emotional–behavioral model, individual behavior will be interpreted by cognition, and individual behavior will be guided by emotion, emphasizing the interaction between internal understanding and the external environment [10]. Therefore, adolescent cyberbullying does not occur alone, but is influenced by the regulation of individual cognition and emotions, and by the online environment in which adolescents live. Based on the above theories, some scholars have started to pay attention to the influence of an individual’s paranoid tendencies on aggressive and hurtful behaviors [11].

Among the adverse psychological problems, paranoia is the more prominent psychological issue [12]. Individuals with paranoid tendencies exhibit more radical views and are prone to hostility toward others. People with paranoid tendencies tend to have biases and cognitive biases when confronted with specific events [13]. It has been noted that adolescents who possess such cognitive biases have an increased frequency of committing aggressive behaviors [14]. Individuals with paranoid tendencies have been reported by scholars to exhibit the following characteristics [12]: being suspicious of others or having more paranoid views, being pessimistic by nature, being easily jealous, being stubborn, and being efficiently hostile towards others [15]. The insecurity and mistrust generated by paranoid tendencies dictate that individuals compete to achieve their goals when faced with conflicting choices. Excessive suspicion and hypersensitivity can exacerbate misperceptions of bystanders and distrust of the surrounding environment, which may lead to higher levels of passive aggression in individuals with paranoid tendencies [11]. Although some scholars have focused on the link between individual paranoid tendencies and aggression, few have examined the relationship between paranoid tendencies in cyberbullying and cyber attacks.

In addition, adolescents are in a stage of rapid physical and mental development and learning ability. Therefore, their cognitive abilities increase, and the emotion–cognition–behavior relationship changes at this stage. During this period, adolescents’ physical and psychological development is unbalanced, their psychology is not fully mature, and their self-regulation ability is weak. Additionally, moral disengagement, as a cognitive variable, can be dysfunctional in the individual’s intrinsic ethical standards under environmental oppression [16]. It has been suggested that moral disengagement can predict adolescent aggression and bullying [17]. Moral disengagement is a specific cognitive tendency that individuals develop that invalidates their internal ethical standards, reducing guilt and shame for committing immoral acts [18]. Bandura, the originator of moral disengagement, argues that whether individuals engage in moral behavior is influenced by both moral mechanisms [19], which reinforce moral standards ethically drive individuals to behave morally, and moral disengagement, which lowers moral standards and lowers ethical standards to induce individuals to behave immorally [20]. Based on cognitive-behavioral theory, some scholars have also focused on the influence of negative emotional tendencies, paranoid personality tendencies, and paranoid psychological tendencies on individuals’ moral disengagement [21]. As well as this, there is research on how moral disengagement, as a cognitive disposition, affects aggressive behavior [22]. Some studies even point out that high levels of moral disengagement predict more indirect bullying [23]. Future studies, however, have yet to examine all three together. In particular, they have not examined moral disengagement as a mediating variable to explore how it plays a role in how paranoid tendencies influence adolescent cyberbullying. Therefore, there is a need to link paranoid tendencies and moral disengagement together to examine the role of adolescent cyberbullying.

### 1.1. The Relationship between Paranoid Tendencies and Cyberbullying

Relevant studies have shown that the formation of paranoid tendencies is closely related to an individual’s cognition. When individuals lack proper cognition of things or have attribution bias and thinking bias, they will develop a paranoid view of events [24]. In a prisoner’s dilemma experiment, Tone et al. found that people with paranoid tendencies are biased toward distrustful competitive choices [25]. In particular, they feel anxiety when others achieve a certain level of honor. The insecurity and mistrust of high paranoia determine that they will compete to achieve their goals in the face of conflicting choices [24]. In the study of paranoid tendencies and bullying, psychologists have argued that higher paranoid tendencies lead to aggressive behavior [14], i.e., individuals with high paranoid tendencies protect themselves from negative emotions by attacking others when they experience frustration and failure. A meta-analysis of children and bullying found that paranoid tendencies and stubborn-mindedness positively predicted bullying behavior [26]. In terms of the relationship between paranoid tendencies and aggression, studies on secondary school students have also shown that paranoid and sensitive personalities are positively associated with aggressive behavior [27]. Similarly, some studies have found that individuals with high sensitivity and anxiety are more likely to commit bullying against others online [28]. Additionally, there is experimental evidence that paranoia positively affects aggression and is more likely to occur in adolescent groups [11]. However, little research has focused on individuals with paranoid tendencies committing cyber bullying. Still, it is known from previous studies that individuals with paranoid tendencies are more aggressive and often engage in bullying behaviors against others. For this reason, we propose Hypothesis 1.

**Hypothesis 1.**  *Paranoid tendencies may be associated with cyberbullying in adolescents.*

### 1.2. The Mediating Role of Moral Disengagement

Moral disengagement refers to the cognitive tendency of individuals to self-justify their unethical behavior [16]. Moral deference is an important explanatory mechanism for the formation of unethical behavior in organizations [18], mainly through moral justification, responsibility transfer, and distraction to isolate the causal relationship between unethical behavior and its consequences, thus freeing individuals from guilt, shame, and conscience [21], making moral deference an important antecedent variable of unethical behavior [29]. Meanwhile, negative life experiences and negative psychological emotions are essential factors that enhance moral disengagement [17]. First, paranoid personality tendencies, such as negative psychological emotions, can impact individuals’ moral disengagement and induce unethical behavior [30]. Specifically, the more severe the paranoid behavior or paranoid mindset of college students or adolescent groups, the more likely they are to engage in moral disengagement when they engage in immoral behavior by, for example, defending their morality and attributing blame to external parties [31]. This is a side effect of the fact that personality traits such as paranoia can impact individuals’ perceptions of moral disengagement. Second, a large body of research confirms that moral disengagement significantly and positively predicts aggressive behavior in adolescents [32]. Moral disengagement is significantly and positively related to online deviant behavior, and can completely predict online aggressive and deceptive conduct [33]. This is because of the virtual nature of the online environment, where individuals lack face-to-face communication in the process of online communication, which leads to a lack of moral cues, resulting in a weakened moral consciousness, a consequent lowering of ethical standards, and a higher level of moral disengagement, showing more online deviant behaviors. The survey shows that the more bullied adolescents are offline, the more likely they are to attribute their cyberbullying behavior to the fact that they first suffered from others [21]. Thus, they make a disengagement when bullying others [22], i.e., rationalizing their aggressive behavior through moral justifications, meaning behavior that seems reasonable and justified, and thus committing more bullying behaviors against others online [23]. Bullying is a deliberate attempt by the strong to harm the weak, and the power imbalance between the two parties makes it immoral [34]. Therefore, this study proposes Hypothesis 2.

**Hypothesis 2.**  *Moral disengagement has a mediating effect on the influence of paranoid tendencies on adolescent cyberbullying.*

**Figure 1:**
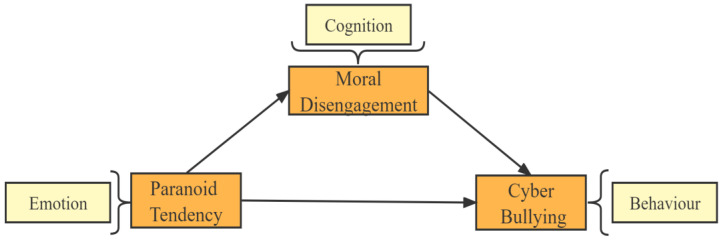
Theoretical framework diagram.

Compared to previous studies that have focused primarily on bullying, fewer studies consider cognition and emotion together in bullying, mostly focusing on cognition alone or emotion alone [25]. Moreover, they have only linked bullying behavior to specific environments in isolation [29]. However, based on cognitive-behavioral theory, it is known that individuals’ behaviors do not occur in isolation and are influenced by cognition and emotions. Additionally, scholars’ research on cyberbullying has focused less on the influence of paranoia as an emotional change, and even less on combining moral disengagement as a cognitive variable. Based on cognitive-behavioral theory, a mediating model is constructed by integrating adolescent cyberbullying into the emotion–cognition–behavior model, introducing the emotional variable of paranoid tendencies and the cognitive mechanism of moral disengagement. The influence of paranoid tendencies and moral disengagement on adolescent cyberbullying behavior was examined. The following is a diagram of the theoretical framework for this study (Figure 1).

## 2. Materials and Methods

### 2.1. Participants and Procedure

This study aimed to examine (1) whether paranoid tendencies influence adolescent cyberbullying behavior, and (2) the mediating mechanism of moral excuses in the influence of paranoid tendencies on adolescent cyberbullying behavior. The empirical data were collected through the online survey platform “Questionnaire Star.” An online questionnaire link was developed and shared with adolescents in six major cities in China, including Shenyang, Beijing, Tianjin, Shanghai, Xiamen, and Xi’an, defining respondents as adolescents in their first to third year of high school. The data covered important cities in eastern, northern, southern, and central China, reflecting the comprehensive coverage of the research scope. The study’s purpose, use, and significance were explained in the first part of the questionnaire. It was emphasized that all contents were confidential and used for scientific research only. The second part included the contents of the Basic Personal Profile and Basic Family Profile questionnaire, the Cyberbullying Scale, the SCL-90 Paranoia Subscale, and the Moral Excuses Scale. To ensure the authenticity of the responses, the participating adolescents were set to take more than five minutes to fill out the questionnaire. A total of 2203 respondents were recruited, and the survey generated a calculation of 1519 complete and valid data analysis response rates of 68.95%. Of these, 770 were boys (50.70% overall) and 749 were girls (49.30% overall); 373 were students in rural areas (24.56% overall), and 1146 were students in urban areas (75.44% overall); the mean age of the subjects was 13.26 ± 1.26 years. The questionnaire was administered with the teachers’ and students’ informed consent. Consent was obtained from the parents of the adolescents in this study. The Academic Ethics Committee of Bohai University and Northeastern University approved the study.

### 2.2. Research Tools

#### 2.2.1. Cyberbullying Scale

A cyberbullying subscale developed by Betts and Spenser was used [35]. There are 18 questions. The questions were primarily designed to represent the number of times an individual committed cyberbullying. The scale is scored on a 4-point scale from 1 “never” to 4 “more than five times”. Higher scores represent higher levels of cyberbullying perpetrated by the individual. In this study, the Cronbach coefficient for this subscale was 0.964, and the cyberbullying scale KMO value was 0.989.

#### 2.2.2. SCL-90 Paranoia Scale

The Paranoia Subscale of the SCL-90, a 6-item questionnaire, was developed around the essential characteristics of paranoid thinking [36]. A score of 12 or more on this subscale indicates that the individual is paranoid and more suspicious and hostile; a score of 6 or less means that the individual is not paranoid, with a total score of 24. The higher the score, the more paranoid the individual is, showing projective thinking and delusions. The lower the score, the less likely the individual is to think in extremes. In the study, the Cronbach coefficient for this subscale was 0.954, and the KMO value for the paranoia subscale was 0.943.

#### 2.2.3. Moral Disengagement Scale

A revised Chinese version of the Civic Moral Disengagement Questionnaire was used by Gini and Pozzoli, with 32 questions [37]. The scale was scored on a 5-point scale, ranging from “totally disagree”, “relatively disagree”, “not sure”, “relatively agree”, and “totally agree”. The higher the score, the higher the level of moral disengagement. In this study, the Cronbach coefficient for this scale was 0.987, and the KMO value for the moral deference scale was 0.996.

### 2.3. Data Analysis

The study was analyzed using SPSS software, and the standard method deviation test, descriptive statistics, regression analysis, correlation analysis, and mediating effect test were performed sequentially.

## 3. Results

### 3.1. Common Method Bias

The research method used in this study included a self-statement scale with possible standard method bias, which was tested using Harman’s one-way method. All questions were subjected to exploratory factor analysis. The results revealed that the KMO value was 0.92, and the *p*-value of Bartlett’s spherical test was less than 0.001, where the variance explained by the first factor was 32.92%, which was below the critical criterion of 40%. Thus, there was no common severe method bias in this study [38].

### 3.2. Regression Analysis of Relevant Variables

Based on the classification results, the effects of demographic variables on paranoid tendencies, moral disengagement, and cyberbullying were examined. Multinomial logistic regressions were conducted with paranoid direction, moral disengagement, and cyberbullying potential as dependent variables and gender, place of origin, and only child as independent variables, respectively, to obtain Odds Ratio coefficients (also known as ratio ratios), with OR coefficients reflecting the relative effects of different levels of the independent variables [39]. As shown in Table 1, in terms of gender, a higher proportion of persons experienced moral disengagement in the group of boys compared to girls, while girls had a higher proportion of paranoid tendencies than boys, and there was no gender difference in cyberbullying; in terms of place of origin, there was no significant difference in the distribution of persons between students from rural and urban areas in terms of paranoid tendencies, moral disengagement, and cyberbullying; in terms of single children, non-single children tend to be more likely to engage in cyberbullying than single children.

### 3.3. Descriptive Statistics

Descriptive statistics and Pearson correlation analysis were first conducted to test the relationship between the study variables and the results presented in Table 2. In line with expectations, cyberbullying among adolescents was significantly and positively associated with paranoid tendencies (*p* < 0.01), i.e., adolescents with paranoid tendencies would commit cyberbullying more frequently. Second, moral disengagement was significantly and positively associated with both paranoid directions and cyberbullying (*p* < 0.01), representing that moral disengagement is positively related to adolescent cyberbullying, a result that paves the way for the subsequent verification of the mediating effect of moral disengagement.

### 3.4. Intermediary Model Testing

According to the mediation test procedure proposed by scholars [40], after controlling for gender and place of origin, in equation 1, paranoid tendency significantly and positively predicted cyberbullying (β = 0.31, *p* < 0.001); in equation 2, paranoid tendency significantly and positively predicted moral disengagement (β = 0.24, *p* < 0.001); in equation 3, both paranoid tendency and moral disengagement were included, and the results showed that paranoid tendency significantly and positively predicted cyberbullying (β = 0.25, *p* < 0.001) and moral disengagement (β = 0.26, *p* < 0.001). Further testing using the bias-corrected percentile Bootstrap method revealed a significant mediating effect of moral disengagement between paranoid tendencies and cyberbullying, with ab = 0.11, SE of 0.01, 95% confidence interval [0.08, 0.14], and a mediating effect of 20.50% of the total effect—see Table 3.

**Figure 2:**
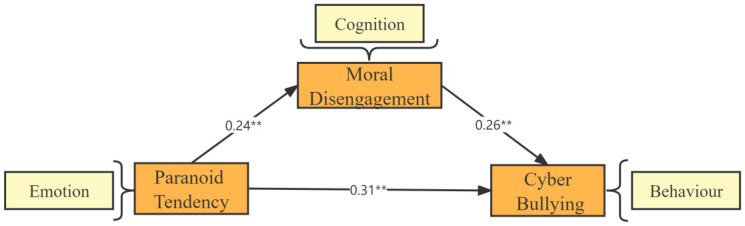
Intermediary effect diagram.

As shown in Figure 2, firstly, the direct effect of paranoid tendencies affecting cyberbullying is significant and β = 0.31, *p* < 0.01, representing that individuals with paranoid tendencies positively influence cyberbullying. Second, moral disengagement also positively affected paranoid trends affecting cyberbullying. In particular, the process of paranoid movement influencing moral disengagement was significantly positive and β = 0.24, *p* < 0.01. Moreover, the process of moral disengagement influencing cyberbullying behavior was also very positive and β = 0.26, *p* < 0.01. The results of this model confirmed the conjecture and application of the emotional–cognitive–behavioral theory model in the previous paper [32].

## 4. Discussion

Based on a review of the relevant literature, some scholars have pointed out that cyberbullying and aggression are somewhat linked [41]. When moral disengagement is mediating, it further increases personal attacks and positively influences individuals committing cyber bullying. Another study has pointed out that paranoia is slightly related to emotions [15]. No scholars have been found who incorporate the three into a mediating model for study, an important empirical innovation of this paper. Therefore, this paper examines the direct effect of paranoid tendencies on cyberbullying behavior by constructing a mediation model, while exploring the mediating role of moral disengagement therein.

### 4.1. The Relationship between Paranoid Tendencies and Cyberbullying

After mediating effects analysis, it was concluded that hypothesis one of this paper holds that paranoid tendencies positively correlate with cyberbullying. This may be because paranoid tendencies cause one to be prone to resentment and to attempt revenge, aspects inherently associated with aggressive behavior [27], so paranoid tendencies may directly increase the probability of cyberbullying occurring. However, paranoia is typical in psychology, with perhaps 1/3 of the general population tending to think paranoid thoughts [13]. Paranoia can cause individuals to have negative emotions and perceptions and can also hurt work and life, so groups with paranoid tendencies should be of concern [12]. It has been concluded that adolescents with a propensity for paranoid thinking are prone to paranoid tendencies and find it difficult to get along with others in a friendly manner. It has been shown that the population of adolescents with paranoid tendencies positively and significantly predicts the frequency of their cyberbullying perpetration [42]. This may be because paranoid thinking tends to make adolescents behave paranoid [43], thus increasing adolescents’ aggressive behavior, which is consistent with previous studies [44].

### 4.2. The Mediating Role of Moral Disengagement

The results of the correlation analysis concluded that hypothesis two of this paper holds. The study showed that cyberbullying and paranoid tendencies were significantly correlated. Paranoid tendencies had a significant positive predictive effect on cyberbullying, with moral inference as a partial mediator, which is consistent with previous investigations [28]. Paranoia causes individuals to have negative emotions and cognitions, and can also hurt work and life, so this may be one of the reasons why emotions are not significantly regulated [27]. Because paranoid tendencies inherently carry negative emotions and cognitions, this leads to a direct effect of paranoid tendencies on cyberbullying.

Adolescence is the transition period from childhood to adulthood. Adolescents’ unbalanced physical and mental development at this stage and their weak self-regulation ability lead them to face psychological crises as well [43]. For example, during this period, adolescents are no longer willing to rely on the help of adults. They expect to think and solve problems independently, forming a relatively stable self-awareness and self-image [42]. However, adolescents still need to fully mature and have yet to learn to think things through, and they are more impulsive than adults in handling things. Emotional expressions are also extreme, with intensified changes of mind, and rebelliousness [43]. Second, adolescents with paranoid personalities are more likely to be influenced by their own negative emotions, leading to bullying behaviors [22]. The results of this study also demonstrate that increased levels of moral disengagement can make individuals more likely to commit bullying behaviors. When adolescents engage in cyberbullying, individuals may have negative experiences (i.e., adverse effects caused by inconsistent knowledge and actions) within themselves because their moral code is challenged [34]. Additionally, to mitigate this negative experience, individuals trigger their internal moral excuses mechanism, i.e., rationalizing their behavior by blaming their behavior on the bully themselves [44]. This may be because paranoid tendencies and moral disengagement as a mental tendency will directly or indirectly impact externally problematic behavior, and are not as much affected by age [41]. On the other hand, emotion is a subjective response to a specific event and has relative stability [5]. Previous studies have focused on a single variable, used regression models for studies in terms of models, and have used cognitive-behavioral theories less often in terms of ideas. Still, this paper interacts with models and approaches to enhance the innovation of the article. In the future, research can further explore the basis of people committing cyberbullying to reduce the perpetration of cyberbullying.

### 4.3. Practical Implications

First, it is essential to conduct the study with adolescents as the research object [34]. In the puberty stage, adolescents undergo significant physical and mental changes. Second, the secretion of hormones brings disorder to emotions, and combined with Erikson’s theory of psychological development, adolescents in this period begin to appear independent, their social range is expanded, paranoid psychological tendencies occur more easily due to different role switching, and immoral or irresponsible behaviors can more easily arise in the online environment [45]. Therefore, a reasonable regulation of emotions and raising moral awareness in adolescent groups will reduce the likelihood of teenage conflict behaviors, and peer interaction and teacher–student interaction problems [46].

Theoretically, this study starts with adolescent cyberbullying to answer how paranoid emotional tendencies can produce cyberbullying behaviors by increasing individuals’ moral disengagement cognitions, and thus cyberbullying. When groups of adolescents chronically have negative emotions, paranoid moods, and paranoid overreaching psychological states, they may activate moral push-off mechanisms that briefly disable moral self-regulatory functions, and thus may engage in cold-eyed bullying [47]. This further validates the validity of ethical abdication theory in explaining ethical misconduct represented by “bystander indifference”. At the practical level, this study verifies the impact of paranoid tendencies and moral excuses on cyberbullying. This reminds educators to be aware of negative subcultures within the student body, and alerts to the erosion of students’ minds. The mediating role of moral excuses also suggests that campus moral educators can start with moral education to strengthen students’ moral “resistance” and increase their “immunity” to harmful norms, and in this way, cut off the path of hostile environments to individuals.

## 5. Limitations

The present study still needs to improve. First, this study limits the paranoid emotions and moral awareness of individuals involved in cyberbullying to the level of individuals with paranoid psychology. However, realistic cyberbullying situations are filled with participants with multiple personality differences, from bullies with typical personalities and emotions to bullies with paranoid feelings, so the comparative study of surfaces is also the focus of future research, rather than focusing only on the moral factors of groups with paranoid tendencies. Second, limited by the sensitive and virtual nature of cyberbullying, this study was unable to construct realistic cyberbullying situations and only explored the role of emotions and moral push-off perceptions in cyberbullying through questionnaire collection and empirical modeling; therefore, future research needs to add in-depth interviews and conduct experimental studies of bullying behaviors. In addition, it remains necessary for future research to consider further updates to the research paradigm to suppress the effects of social approval effects. Third, this study used a self-assessment scale with self-reporting by the subjects. Even though the principle of confidentiality was repeatedly emphasized during the administration, the response questionnaire may still be influenced by the social expectations of the issues. Further validation can be carried out with other research paradigms to draw more accurate conclusions.

## 6. Conclusions

Based on the findings of this paper, it can be concluded that cyberbullying indeed exists among adolescents, and individuals with paranoid tendencies are more likely to commit cyberbullying behaviors. It is recommended to alleviate adolescent anxiety and tension and improve paranoia at the family and social levels.

At the family level, parents should always be aware of the psychological trends of their teenagers. At the same time, parents have to keep themselves informed of their teenagers’ online situation. When a teenager is in an unpleasant or difficult situation on the Internet, they should give the teenager enough care. Parents should promptly guide their children on what proper online behavior entails. Suppose they find that their children are involved in related cyberbullying incidents. In that case, parents can take several measures: First, encourage their child to speak to a trusted adult about these negative experiences [6]. Ignore the person’s name-calling or ridicule it for the time being so as not to cause undue psychological stress. Secondly, encourage their child to write about the time, place, and content of the bullying to vent their emotions on the one hand, and provide evidence on the other, so that parents can look at the website where the bullying occurred and the process, and ask the administrator of that website to immediately adjust the permission settings to keep the other person from continuing to harass their child’s online activities [48]. Parents should ensure that their child feels safe, offer unconditional support, and ask for ideas to improve the situation.

At the social level, help is sought from public social intervention. First, schools can strengthen students’ cyberethics education by setting up special groups for cyberbullying prevention, and encouraging and properly guiding students to set the proper standards of moral judgment [41]. Schools can also try to combine cyberbullying and traditional school bullying by adding cyberbullying to the conventional mental health curriculum, with psychology teachers intervening in students’ cyberbullying behavior. At the same time, they should focus on the combination of theoretical knowledge and practical behavior, and practical consideration of the actual lives of students, so that moral education theory has a root to follow and really makes its way into the hearts of students. Adolescents are in the developmental stage and are highly malleable, so paying attention to model education and encouraging students to form correct behavioral models to establish valid worldviews, outlooks on life, and values is essential. In addition, teachers should guide students to learn self-reflection and improve their self-reflection skills, which are extremely important for cultivating positive morality. This is expected to provide society with a more comprehensive understanding of the actual state of cyberbullying, and to lead people to be aware of the influencing factors of cyberbullying behavior. As for moral disengagement, it is of practical significance and importance to strengthen the ideological and ethical education for the youth population, to further cultivate their cyber moral values, and reduce the frequency of cyberbullying behaviors committed.

Finally, let us take the above two points together. We can find that if we intervene in youth cyberbullying from the perspective of moral excuses, we need to start from both cognitive-emotional regulation and behavioral shaping; in terms of cognitive-emotional, both schools and families should correct the wrong beliefs of individuals with high moral excuses, help them establish correct moral value judgment standards and a sense of shame in line with usual social norms, so they do not wantonly justify immoral behaviors [44]. In terms of behavior, both families and schools should adopt a “zero tolerance” attitude towards bullying, help high moral-deferring individuals by encouraging moral behaviors, reducing the frequency of using retaliatory means in conflict situations, and gradually helping them to improve their moral judgment and behavior strategies in ethical conditions. Therefore, in social life, we should always pay more attention to these adolescent populations, grasp the right psychological counseling skills and corresponding measures, and use appropriate methods to help these students with paranoid thinking tendencies to stay out of trouble. Thus, cyberbullying can be reduced, which is conducive to building a harmonious online environment.

## Figures and Tables

**Table 1:** Regression analysis of relevant variables.

	Variable	OR	CI (95%)
Cyberbullying	Gender (with a female as reference)	0.77	0.59–0.98
	Place of origin (rural as a reference)	0.75	0.45–0.95
	Whether they are a single child (non-single child as a reference)	0.92 **	0.59–1.38
Paranoid Tendency	Gender (with a male as reference)	0.34 *	0.23–0.86
	Place of origin (rural as a reference)	0.33	0.14–0.79
	Whether they are a single child (non-single child as a reference)	0.36	0.28–1.79
Moral Disengagement	Gender (with a female as reference)	0.54 **	0.12–1.11
	Place of origin (rural as a reference)	0.17	0.04–1.21
	Whether they are a single child (non-single child as a reference)	0.45	0.46–1.76

Note: ** *p* < 0.01, * *p* < 0.05.

**Table 2:** Descriptive statistics and correlations.

	M ± SD	Cyberbullying	Paranoid Tendency	Moral Disengagement
Cyberbullying	1.21 ± 1.21	1		
Paranoid Tendency	1.50 ± 0.47	0.31 **	1	
Moral Disengagement	1.41 ± 2.79	0.26 **	0.25 **	1

Note: ** *p* < 0.01, * *p* < 0.05.

**Table 3:** Analysis of the mediating effect between paranoid tendencies, moral disengagement, and cyberbullying behavior.

Predictor Variable	Equation 1: Cyberbullying		Equation 2: Moral Disengagement		Equation 3: Cyberbullying	
	*β*	*t*	*β*	*t*	*β*	*t*
Gender	0.06	4.16 **	0.03	1.34 **	−0.05	−5.15
Place of origin	0.00	0.25	0.02	1.46	0.01	0.53
Paranoid tendency	0.31	21.19 **	0.24	14.94 **	0.25	16.26 **
Moral disengagement					0.26	15.34 **
*F*	114.41 **		62.24 **		146.24 **	
*R^2^*	0.11		0.07		0.13	

Note: ** *p* < 0.01, * *p* < 0.05.

## Data Availability

The raw data supporting the conclusions of this article will be made available by the authors without undue reservation.
